# Slot-Based Antimicrobial Low-Cost Non-surgical Periodontal Therapy in the Management of Periodontitis: A Case Series and Narrative Review

**DOI:** 10.7759/cureus.102194

**Published:** 2026-01-24

**Authors:** Rohit Mishra, Varsha Choubey, Elashri Chatterjee, Sukirti Singh, Priyanshi Wasalya, Dhairya Jain, Shalini Mishra, Anshul Gulati, Paramjeet K Chhabra, Sheewali Saggar, Nikita Saini

**Affiliations:** 1 Periodontics and Implantology, Hitkarini Dental College and Hospital, Jabalpur, IND; 2 Periodontics and Implantology, Private Practice, Jabalpur, IND; 3 Dentistry, Civil Hospital Ranjhi, Jabalpur, IND

**Keywords:** adjunctive periodontal therapy, antimicrobial therapy, chemical plaque control, periodontal pathogens, periodontitis, slot therapy

## Abstract

Periodontitis is a chronic infectious and inflammatory disease characterized by progressive destruction of the periodontal ligament and alveolar bone. While scaling and root planing (SRP) remains the cornerstone of non-surgical periodontal therapy, mechanical debridement alone may be inadequate in cases with deep periodontal pockets and persistent microbial reservoirs. In the context of periodontitis, “slot-based therapy” refers to a comprehensive anti-infective treatment strategy based on pathogen-directed antimicrobial principles and low-cost periodontal care concepts, combining mechanical debridement with adjunctive antimicrobial measures in a simplified, cost-effective, and evidence-based approach, particularly suited to resource-limited clinical settings.

This article presents a case series of three patients with localized and generalized periodontitis who were managed using non-surgical slot therapy protocols. All patients exhibited varying degrees of gingival inflammation, plaque accumulation, and compromised periodontal health at baseline. Treatment involved ultrasonic SRP supplemented with adjunctive antiseptic-based antimicrobial strategies, including povidone-iodine subgingival irrigation and dilute sodium hypochlorite rinses for maintenance, with systemic antimicrobials prescribed selectively based on clinical indication. Clinical follow-up demonstrated a consistent reduction in gingival inflammation, improved gingival contour, and enhanced periodontal health across all three cases. No adverse effects were observed during the treatment or follow-up periods. The outcomes observed in this case series support the clinical utility of slot therapy as a pragmatic adjunct to conventional non-surgical periodontal treatment, offering a viable alternative to technology-dependent modalities while achieving meaningful clinical improvement.

## Introduction

Periodontal diseases are highly prevalent worldwide, affecting an estimated 20-50% of the global population across both developed and developing countries [1,2]. Periodontitis, a chronic inflammatory condition, impacts nearly half of the adult population, with severe forms affecting approximately 10-15% of individuals, underscoring its substantial public health relevance across adolescents, adults, and older age groups [1,2]. Clinically, the disease is characterized by episodic inflammation, periodontal attachment loss, and alveolar bone destruction, ultimately leading to tooth loss if untreated [3]. Epidemiological studies have consistently demonstrated a strong association between periodontitis and systemic conditions such as diabetes mellitus, cardiovascular disease, and adverse pregnancy outcomes [4,5].

The clinical presentations observed in the three patients included in the present case series, ranging from localized severe gingival inflammation to generalized periodontal involvement, reflect this well-documented disease burden and its variable expression across individuals.

The pathogenesis of periodontitis involves the accumulation of a dysbiotic dental plaque biofilm that triggers a destructive host inflammatory response, ultimately leading to periodontal tissue breakdown [3,6,7]. The disease is associated with, and likely caused by, a complex interaction between virulent bacterial species, various mammalian viruses, and pro-inflammatory host responses. The most prominent putative periodontal pathogens include gram-negative anaerobic rods and herpesviruses [3,4,8]. These pathogenic mechanisms provide a biological explanation for the pronounced gingival inflammation, plaque accumulation, and tissue breakdown observed at baseline in all three cases presented in this report.

Despite the proven effectiveness of scaling and root planing (SRP), several studies have demonstrated that mechanical therapy alone may be insufficient to eliminate pathogenic microorganisms from deep periodontal pockets, furcation areas, and root concavities [9]. This limitation is clinically relevant, as residual microbial reservoirs may contribute to persistent inflammation even after conventional therapy. In the present case series, initial clinical findings suggested that mechanical debridement alone might be inadequate to fully address the inflammatory burden, thereby justifying the use of adjunctive antimicrobial measures.

These limitations have encouraged the use of adjunctive antimicrobial strategies, which form the conceptual foundation of the approach referred to in this manuscript as slot therapy. This approach emphasizes accessibility, affordability, and effective microbial suppression by integrating mechanical debridement with cost-effective antiseptic and antimicrobial agents. The term “slot therapy” is used descriptively rather than to denote a formally established periodontal treatment modality and reflects a simplified, antimicrobial-based non-surgical periodontal treatment concept derived from the pathogen-directed and low-cost periodontal therapy principles proposed by Slots [4]. The three cases presented in this article were managed in accordance with these principles, providing a clinical illustration of slot therapy as a pragmatic, non-surgical approach to periodontal disease management, particularly relevant in settings where access to advanced periodontal care may be limited.

## Case presentation

In all the cases, baseline periodontal assessment demonstrated generalized periodontal pocketing, bleeding on probing, and poor plaque control. All patients were systemically healthy, with no reported medical comorbidities and no history of systemic conditions known to influence periodontal status.

Case 1

**Figure 1:**
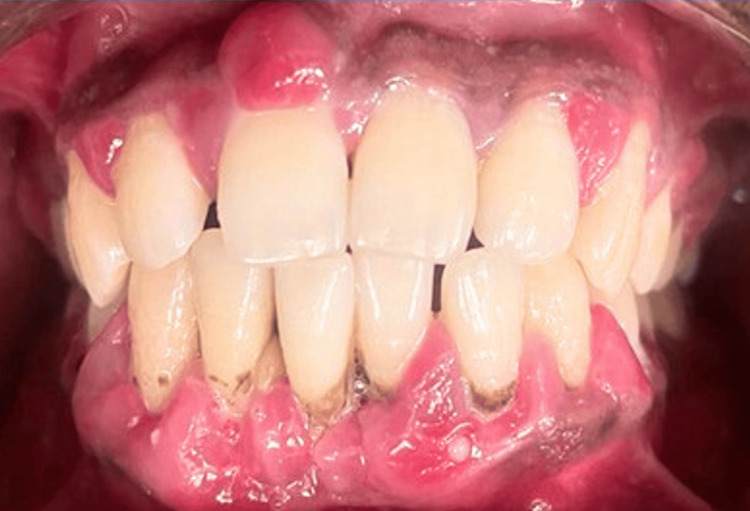
Pre-operative clinical view of Case 1 showing generalized gingival inflammation, erythema, and plaque accumulation consistent with chronic periodontitis.

A patient presented with generalized gingival inflammation, erythema, and visible plaque accumulation consistent with chronic periodontitis. Pre-operative clinical examination revealed inflamed gingival margins, plaque retention, and compromised periodontal health, as shown in Figure 1.

Based on the severity and generalized nature of the disease, the patient was managed using a structured slot therapy protocol consistent with low-cost periodontal treatment principles. On Day 0, treatment included povidone-iodine subgingival irrigation performed prior to ultrasonic scaling, followed by thorough ultrasonic scaling and repeat povidone-iodine subgingival irrigation post-scaling. Adjunctive antiviral therapy with valacyclovir (500 mg twice daily for 10 days) was prescribed. The patient was instructed to perform self-care, subgingival irrigation, and oral rinsing using dilute (0.2%) sodium hypochlorite two to three times weekly as a long-term maintenance measure.

On Day 10, the same sequence of povidone-iodine subgingival irrigation before and after ultrasonic scaling was repeated. Adjunctive systemic antibiotic therapy was prescribed according to patient suitability, consisting of amoxicillin-metronidazole (250 mg of each, three times daily for eight days), in accordance with slot therapy recommendations for advanced or refractory periodontitis.

**Figure 2:**
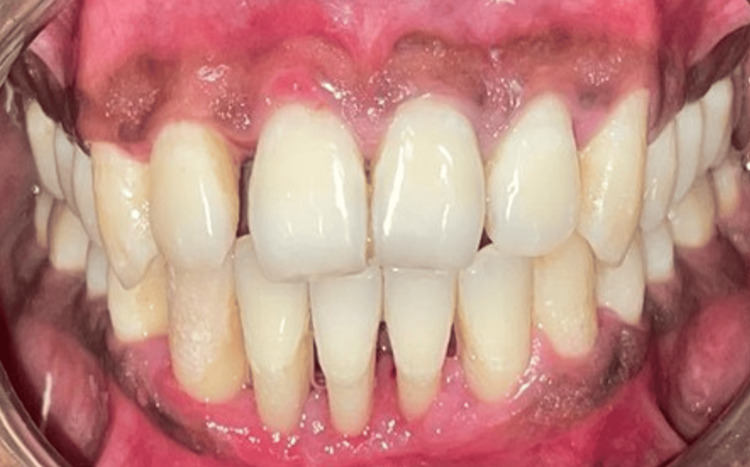
Post-operative clinical view of Case 1 demonstrating marked reduction in gingival inflammation, improved gingival contour, and enhanced plaque control following non-surgical slot therapy.

At the subsequent 10-day follow-up, post-treatment clinical evaluation revealed a substantial reduction in gingival inflammation, with improved gingival contour and better plaque control, as shown in Figure 2.

Case 2

**Figure 3:**
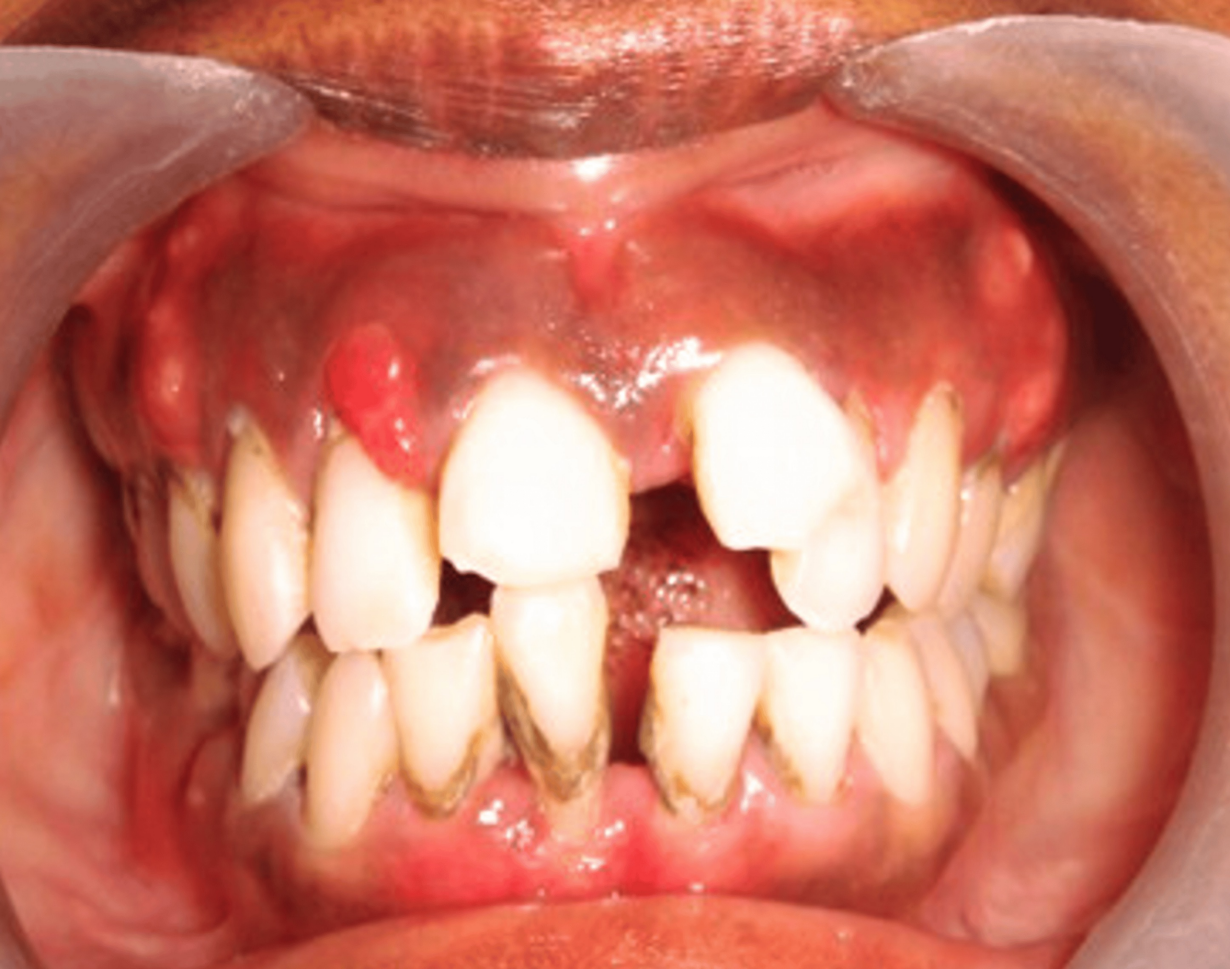
Pre-operative clinical view of Case 2 showing localized severe gingival inflammation in the anterior region with associated plaque accumulation.

This patient presented with localized severe gingival inflammation in the anterior region, accompanied by periodontal pocketing and plaque accumulation. The pre-operative clinical view showed pronounced gingival erythema and edematous tissues, as shown in Figure 3.

In view of the severity of localized periodontal inflammation, the patient was managed using the same structured slot-based antimicrobial non-surgical periodontal treatment protocol applied in Case 1. On Day 0, povidone-iodine subgingival irrigation was performed before ultrasonic instrumentation, followed by comprehensive ultrasonic scaling and repeated povidone-iodine irrigation after debridement. Adjunctive antiviral therapy with valacyclovir (500 mg twice daily for 10 days) was prescribed.

The patient was advised to perform regular self-care, subgingival irrigation, and oral rinsing using dilute (0.2%) sodium hypochlorite two to three times per week as part of the maintenance regimen.

At the Day 10 visit, the same sequence of pre- and post-scaling povidone-iodine irrigation combined with ultrasonic scaling was repeated. Adjunctive systemic antibiotic therapy was administered, consisting of ciprofloxacin-metronidazole (500 mg of each, twice daily for eight days).

**Figure 4:**
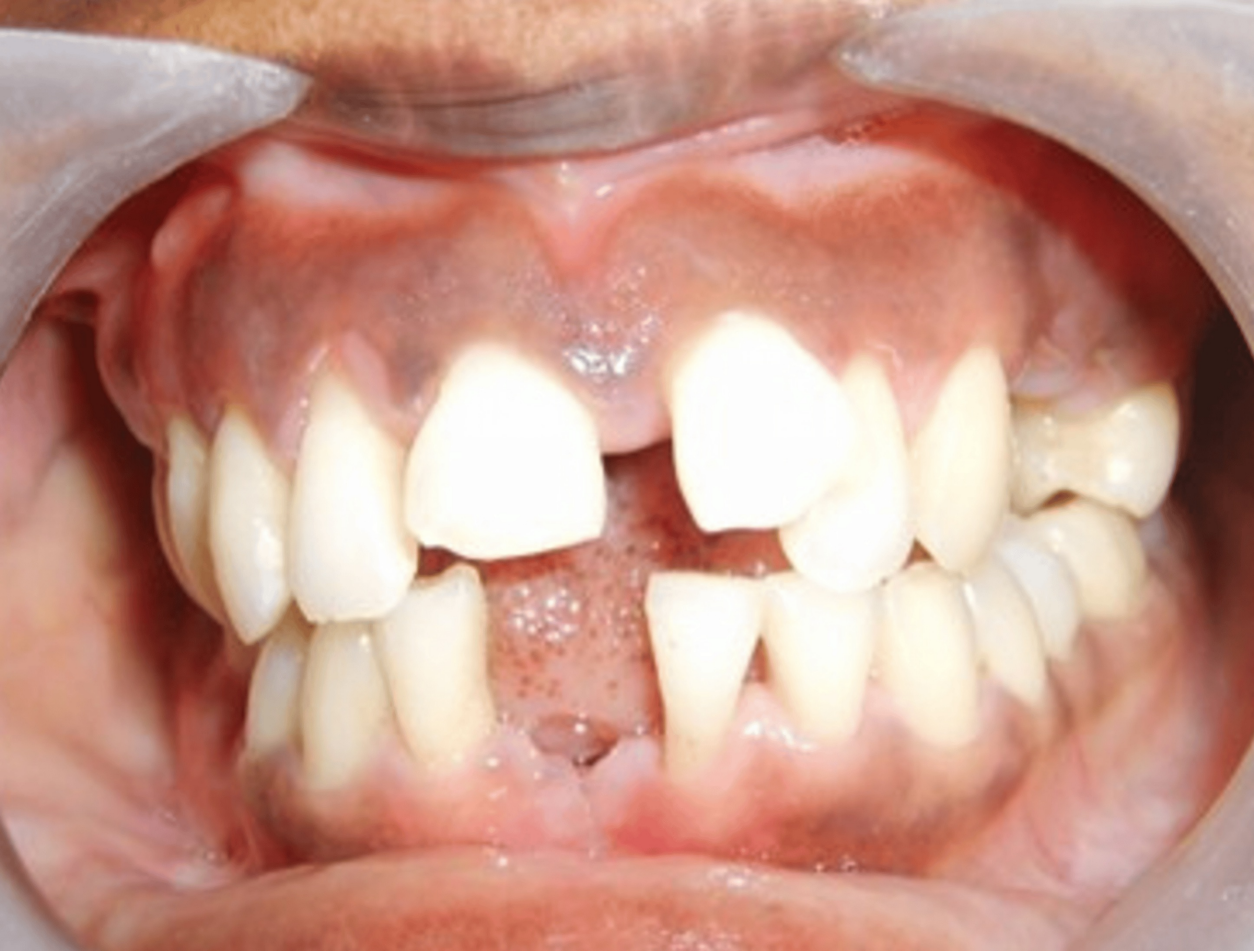
Post-operative clinical view of Case 2 illustrating resolution of gingival inflammation and improvement in periodontal tissue tone following slot therapy.

Post-operative evaluation at the Day 20 visit revealed substantial clinical improvement, including reduced gingival inflammation, improved tissue tone, and overall enhancement of periodontal health, as shown in Figure 4.

Case 3

**Figure 5:**
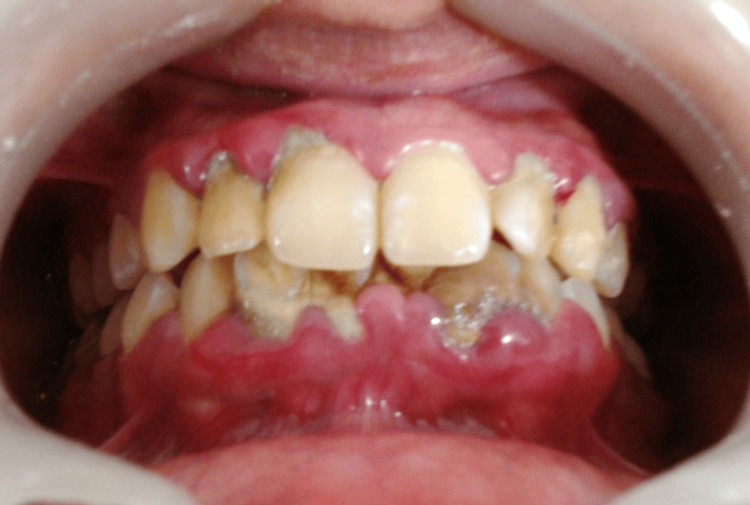
Pre-operative clinical view of Case 3 showing generalized plaque deposits, gingival erythema, and compromised periodontal health.

A patient with generalized periodontal involvement presented with extensive plaque deposits, gingival erythema, and compromised periodontal status. The pre-operative image demonstrated significant inflammation and plaque retention, as shown in Figure 5.

Considering the generalized disease severity, management was carried out using a structured slot-based antimicrobial non-surgical periodontal therapy protocol. On Day 0, povidone-iodine subgingival irrigation was performed prior to ultrasonic scaling, followed by comprehensive ultrasonic scaling and repeated povidone-iodine subgingival irrigation after instrumentation. Adjunctive antiviral therapy with valacyclovir (500 mg twice daily for 10 days) was prescribed.

The patient was instructed to undertake self-care subgingival irrigation and oral rinsing with dilute (0.2%) sodium hypochlorite two to three times weekly as part of the maintenance regimen.

At the 10-day follow-up visit, the same sequence of povidone-iodine subgingival irrigation before and after ultrasonic scaling was repeated. Systemic antibiotic therapy was prescribed according to patient suitability, consisting of amoxicillin-metronidazole (250 mg of each, three times daily for eight days).

**Figure 6:**
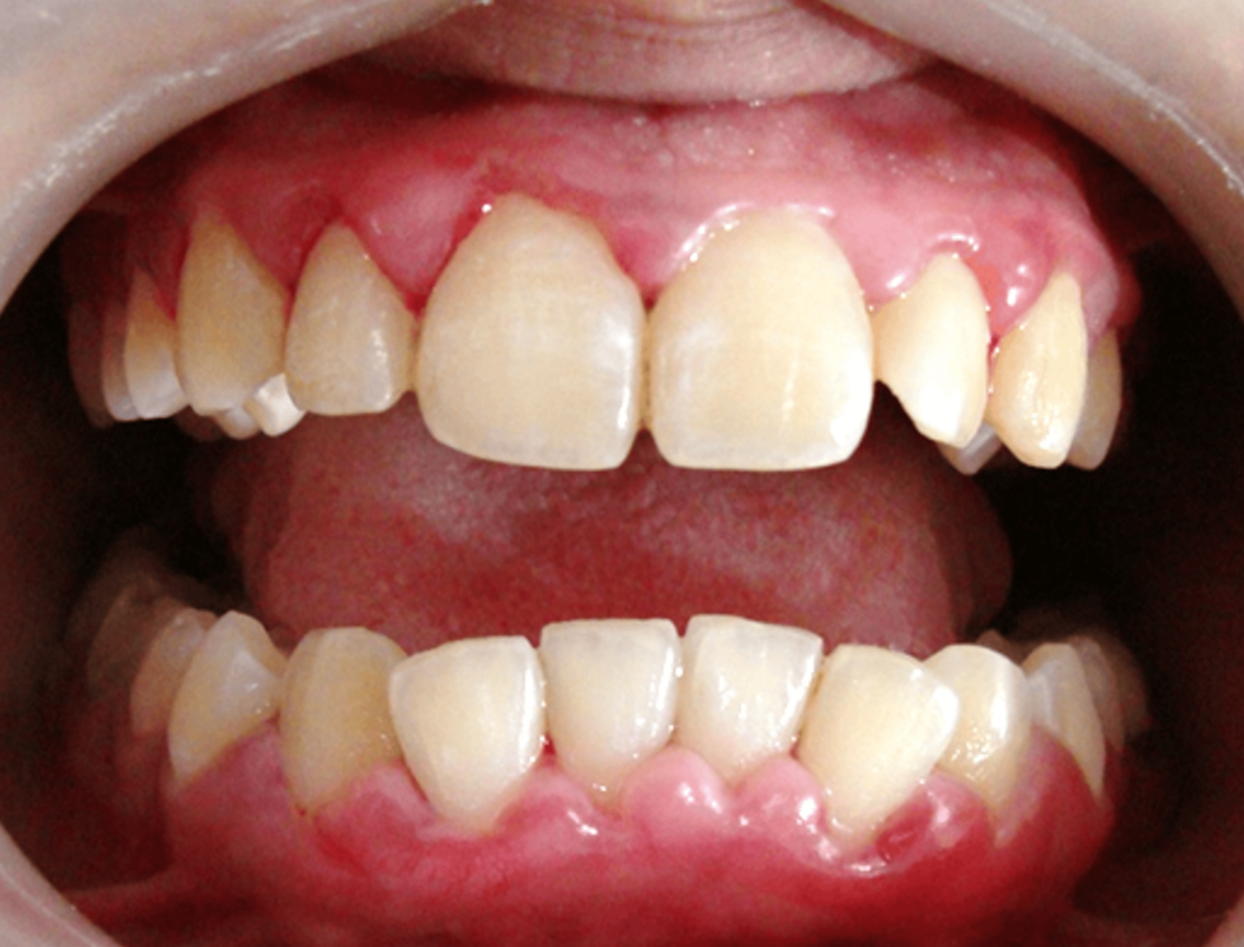
Post-operative clinical view of Case 3 demonstrating improved gingival architecture, reduced inflammation, and better plaque control after non-surgical slot therapy.

At post-treatment evaluation after the next 10 days, the gingival tissues appeared healthier with reduced inflammation, improved gingival architecture, and better plaque control, as shown in Figure 6.

Post-treatment evaluation demonstrated a marked reduction in gingival inflammation and bleeding on probing, with improved plaque control in all cases. The use of antiviral therapy was based on evidence demonstrating the association of herpesviruses and other viruses with periodontal disease activity and tissue destruction, as reported by Slots and colleagues, who emphasized the role of antiviral agents in selected cases exhibiting severe inflammatory periodontal breakdown [8].

## Discussion

Periodontal disease is associated with a shift from a symbiotic microbial ecosystem to a dysbiotic biofilm dominated by gram-negative anaerobic microorganisms. Among these, Porphyromonas gingivalis has been identified as a keystone pathogen due to its ability to modulate host immune responses and promote dysbiosis even at low abundance [10]. In addition to bacterial pathogens, viral agents such as Epstein-Barr virus and human cytomegalovirus have been detected in periodontal lesions and are believed to exacerbate periodontal destruction by impairing local immune defense mechanisms [11]. Furthermore, patients with aggressive or refractory forms of periodontitis frequently exhibit elevated levels of *Aggregatibacter actinomycetemcomitans*, a pathogen capable of invading periodontal tissues and evading mechanical removal [12]. The generalized and localized inflammatory presentations observed across the three cases in the present series are consistent with these established microbiological mechanisms.

SRP remains the gold standard for initial periodontal therapy [9]. Cobb reported significant reductions in probing depth and bleeding on probing following SRP; however, residual calculus and pathogenic bacteria were frequently detected in periodontal pockets exceeding 5 mm in depth [13,14]. Waerhaug further demonstrated that complete calculus removal was achieved in less than half of deep periodontal pockets, even when treatment was performed by experienced clinicians [15].

In the present case series, although non-surgical mechanical debridement formed the foundation of treatment, adjunctive antimicrobial measures were employed to address these known limitations of SRP, particularly in areas exhibiting persistent inflammation at baseline.

Adjunctive non-conventional therapies, including lasers and photodynamic therapy, have been explored to overcome these limitations. However, current evidence does not support the superiority of laser therapy over conventional SRP in the management of chronic periodontitis, as consistent evidence of superior clinical attachment gain remains lacking [16]. Similarly, photodynamic therapy has demonstrated only transient reductions in microbial load when used adjunctively with SRP, limiting its long-term clinical utility [17]. Given these constraints, advanced technology-dependent modalities were not incorporated into the management of the present cases.

Slot-inspired therapy represents a simplified, pathogen-directed periodontal treatment approach derived from antimicrobial therapy principles proposed by Dr. Jørgen Slots, emphasizing mechanical debridement combined with affordable antiseptic and antibiotic agents to suppress periodontal pathogens, particularly in resource-limited settings [4,18]. The clinical protocols applied in all three cases were aligned with this concept, focusing on cost-effective antimicrobial suppression rather than technology-intensive interventions.

Among antiseptic agents, povidone-iodine has demonstrated broad-spectrum antimicrobial activity against gram-positive and gram-negative bacteria, fungi, viruses, and protozoa. Slots and colleagues reported rapid suppression of periodontopathic bacteria following subgingival irrigation with povidone-iodine during periodontal therapy [18]. Hoang et al. further demonstrated that subgingival irrigation with 10% povidone-iodine used adjunctively with SRP resulted in substantial reductions in periodontal pathogens and a mean probing depth reduction of approximately 1.8 mm in deep periodontal pockets [19].

The marked reduction in gingival inflammation and visible clinical improvement observed post-operatively in the present cases reflects these documented antimicrobial benefits when antiseptics are combined with mechanical debridement.

In addition to subgingival irrigation, antiseptics such as sodium hypochlorite and povidone-iodine have been incorporated into ultrasonic scaler cooling sprays to enhance antimicrobial efficacy. The use of sodium hypochlorite or 1% povidone-iodine in scaler coolant has been shown to reduce pathogenic bacterial and viral loads and to minimize aerosolization of infectious agents during periodontal procedures [20,21]. Although microbiological sampling was not performed in the present case series, the favorable clinical tissue responses observed post-therapy are consistent with the documented antimicrobial effects of these adjunctive measures.

Dilute sodium hypochlorite solutions exhibit strong antimicrobial activity against oral biofilms. De Nardo et al. demonstrated that a 0.05% sodium hypochlorite oral rinse significantly reduced supragingival plaque accumulation and gingival inflammation when used alongside mechanical periodontal treatment [22]. Similarly, Lobene and colleagues reported that subgingival irrigation with 0.5% sodium hypochlorite produced a more pronounced and sustained reduction in dental plaque and gingival inflammation compared with water irrigation alone [23]. Histologic evidence further supports its safety and efficacy, with studies demonstrating chemolysis of periodontal pocket epithelium and favorable connective tissue healing [18,24]. The consistent post-treatment reduction in erythema, edema, and plaque accumulation across all three cases aligns with these previously reported clinical and histologic outcomes.

Chlorhexidine remains the most extensively studied antiplaque agent and is effective against supragingival biofilms and oral mucosal microorganisms when used as a 0.12-0.2% mouthrinse [18]. However, its long-term use is limited by adverse effects such as tooth staining and taste disturbance, as well as reduced activity against certain gram-negative organisms [18,25]. Other antiseptic agents, including hydrogen peroxide, hexetidine, phenolic compounds, and quaternary ammonium compounds, demonstrate variable antimicrobial efficacy with recognized clinical limitations [26-28]. Accordingly, long-term maintenance strategies in the present cases emphasized simplified, tolerable antimicrobial measures combined with reinforced oral hygiene practices rather than prolonged reliance on any single agent.

Systemic antibiotics were prescribed in our cases as was clinically indicated, and the use of antiviral therapy was guided by evidence demonstrating an association between viruses and periodontal disease activity and tissue destruction, as reported by Slots and colleagues, who emphasized the role of antiviral agents in selected cases exhibiting severe inflammatory periodontal breakdown [8]. Notably, all three cases demonstrated meaningful clinical improvement following adjunctive systemic antimicrobial therapy, supporting the clinical utility of antiseptic-based slot therapy in appropriately selected periodontal cases.

The present case series thus provides a clinical illustration of literature-supported slot therapy principles, demonstrating that adjunctive antimicrobial strategies combined with mechanical debridement can achieve favorable periodontal outcomes. Long-term periodontal stability remains heavily dependent on effective plaque control and maintenance care, as demonstrated by Axelsson and Lindhe, who reported minimal periodontal breakdown among patients enrolled in structured plaque-control programs [29].

## Conclusions

Slot therapy represents a pragmatic and evidence-based approach to periodontal management by integrating mechanical debridement with affordable and accessible antimicrobial strategies. The three cases presented in this report clinically illustrate that adjunctive antimicrobial measures, when combined with SRP, can result in meaningful improvements in gingival inflammation, plaque control, and overall periodontal health without reliance on advanced technology or routine systemic antibiotic use. The favorable clinical outcomes observed across cases align with existing literature demonstrating the antimicrobial efficacy of antiseptic agents such as povidone-iodine and sodium hypochlorite when used as adjuncts to conventional periodontal therapy. These findings are particularly relevant in resource-limited settings, where cost-effective, simplified treatment protocols may improve treatment accessibility, patient compliance, and long-term disease control.

While slot therapy does not replace conventional mechanical periodontal treatment, it serves as a valuable adjunctive strategy for suppressing residual microbial burden and supporting periodontal stability. Future studies employing standardized protocols, microbiological assessment, and long-term follow-up are warranted to further validate the role of slot therapy in routine periodontal practice.
